# Causes of Death among People Living with AIDS in the Pre- and Post-HAART Eras in the City of São Paulo, Brazil

**DOI:** 10.1371/journal.pone.0114661

**Published:** 2014-12-11

**Authors:** Carmen-Silvia Bruniera Domingues, Eliseu Alves Waldman

**Affiliations:** 1 São Paulo State Program for STDs and AIDS, São Paulo State Department of Health STD and AIDS Referral and Training Center, São Paulo, Brazil; 2 Department of Epidemiology, University of São Paulo School of Public Health, São Paulo, Brazil; Vanderbilt University, United States of America

## Abstract

**Objective:**

We examine the trend in causes of death among people living with AIDS in the city of São Paulo, Brazil, in the periods before and after the introduction of highly active antiretroviral therapy (HAART), and we investigate potential disparities across districts of residence.

**Methods:**

Descriptive study of three periods: pre-HAART (1991–1996); early post-HAART (1997–1999); and late post-HAART (2000–2006). The data source was the São Paulo State STD/AIDS Program and São Paulo State Data Analysis Foundation. Causes of death were classified by the ICD-9 (1991–1995) and ICD-10 (1996–2006). We estimated age-adjusted mortality rates for leading underlying causes of death and described underlying and associated causes of death according to sociodemographic characteristics and area of residence. We used Pearson's chi-square test or Fisher's exact test to compare categorical variables. Areas of residence were categorized using a socioeconomic index. To analyze trends we apply generalized linear model with Poisson regression.

**Results:**

We evaluated 32,808 AIDS-related deaths. Between the pre- and late post-HAART periods, the proportion of deaths whose underlying causes were non-AIDS-related diseases increased from 0.2% to 9.6% (p<0.001): from 0.01% to 1.67% (p<0.001) for cardiovascular diseases; 0.01% to 1.62% (p<0.001) for bacterial/unspecified pneumonia; and 0.03% to 1.46% (p<0.001) for non-AIDS-defining cancers. In the late post-HAART period, the most common associated causes of death were bacterial/unspecified pneumonia (35.94%), septicemia (33.46%), cardiovascular diseases (10.11%) and liver diseases (8.0%); and common underlying causes, besides AIDS disease, included non-AIDS-defining cancers in high-income areas, cardiovascular diseases in middle-income areas and assault in low-income areas.

**Conclusions:**

The introduction of HAART has shifted the mortality profile away from AIDS-related conditions, suggesting changes in the pattern of morbidity, but heterogeneously according to area of residence. There is a need for public policies aimed at adapting health care services to address the new scenario.

## Introduction

Among HIV-infected patients, the introduction of highly active antiretroviral therapy (HAART) changed the patterns of morbidity and mortality, as well as increasing survival, thereby transforming AIDS into a long-term illness [1]. As a result, certain comorbidities assumed greater importance and came to influence the survival of HIV-infected patients. Chief among those comorbidities are hepatitis B, hepatitis C, arterial hypertension, diabetes mellitus, cardiovascular diseases, lung diseases, and non-AIDS-defining cancers (NADCs) [2], [3].

This new pattern of morbidity and mortality can differ across countries. A recent study comparing the cities of Rio de Janeiro, Brazil, and Baltimore, Maryland, USA, in terms of the causes of death among AIDS patients, found that the increase of proportion of deaths in which there was a non-AIDS-related underlying cause was significantly higher in Baltimore [4]. Other studies have shown that such non-AIDS-related deaths are more common among patients over 40 years of age [5] and less so among females, possibly, because the latter have limited access to treatment or show a lower prevalence of risk factors for violent death, cardiovascular disease, and lung cancer [6].

In view of the scenario outlined above, information on causes of death is important and can aid in establishing priorities for public policies to prevent and manage comorbidities [1]. However, few studies have examined the changes in the morbidity and mortality profiles of people living with AIDS in the pre- and post-HAART eras in developing countries. Most such studies have focused exclusively on the post-HAART era [7], [8] or have not been population-based [9]. The objectives of the present study were to describe the changes in the underlying and associated causes of death among people living with AIDS in the city of São Paulo, Brazil, in the periods before and after the introduction of HAART (i.e., 1991–1996 and 1997–2006, respectively) to analyze trends in groups of selected causes of death; and to investigate potential disparities across districts of residence in the 2000–2006 period.

## Methods

This was a population-based descriptive study of people living with AIDS and residing in the city of São Paulo at the time of diagnosis. The city of São Paulo, which has approximately 11 million inhabitants across 96 administrative districts, is the largest and richest city in Brazil. The mean Human Development Index is 0.841 for the city as a whole but varies widely across districts (range, 0.245–0.884) [10]. São Paulo is notable for being more affected by the HIV/AIDS epidemic than has been any other city in Brazil, accounting for approximately 15% of all cases reported in the country [11], [12].

The causes of deaths occurring in the 1991–1995 period and in the 1996–2006 were classified in accordance with the ninth and tenth revisions of the International Classification of Diseases (ICD-9 and ICD-10), respectively, the underlying causes of death having been classified in accordance with the World Health Organization criteria. The remaining causes of death listed on the death certificates were designated associated causes of death. To make the ICD-9 and ICD-10 data comparable, we employed the technique described by Santo & Pinheiro [13], together with the ICD-9/ICD-10 code correspondence table devised by Suárez et al. [14].

The criteria for inclusion in the present study were as follows: having been an AIDS case; having been a resident of the city of São Paulo at the time of diagnosis; having been ≥ 13 years of age at diagnosis; having been registered in the *Base Integrada Paulista de AIDS* (BIPAIDS, São Paulo State Integrated AIDS Database); and having died between January 1, 1991 and December 31, 2006. We excluded HIV-infected individuals who had not developed AIDS. We were able to apply that exclusion criteria because the reporting of cases of HIV infection in the state of São Paulo has been standard practice since 1994, and, as of 2002, that information has been included in the Brazilian *Sistema de Informação de Agravos de Notificação* (SINAN, National Case Registry Database) [15].

In this study, we defined cases of AIDS on the basis of the defining criteria in effect at the time of their diagnoses: the 1987 CDC modified case definition (the 1985 Centers for Disease Control and Prevention definition), revised in 1992, 1998 and most recent revision in 2004 (CDC adapted case definition); the 1992 Rio de Janeiro/Caracas definition (known as the Pan American Health Organization/Caracas case definition); the 1996 exceptional death certificate criterion (mention of AIDS or equivalent term in any field on the death certificate; or any HIV/AIDS-related disease listed as an associated cause of death; and inconclusive epidemiological investigation); or the 1998 definition of a CD4+ T-lymphocyte count <350 cells/mm^3^ [16].

The data sources were: the BIPAIDS, which is a linkage database created (by a deterministic method) from the death database of the São Paulo *Sistema Estadual de Análise de Dados* (SEADE, State System of Data Analysis) and the São Paulo State STD/AIDS Program - SINAN Reportable Diseases Database [17]; and the SEADE database of population estimates (comprising 1991 and 2000 census data for Brazil).

Linking the database of reported AIDS cases with that comprising all-cause mortality data for the state of São Paulo made it possible to identify AIDS-related deaths of individuals whose diagnosis of AIDS had not been reported, dating back to the beginning of the epidemic, the objective being to address and reduce underreporting of AIDS cases in the state. This also allowed us to identify deaths from other causes among the AIDS cases reported to SINAN. These databases are linked on an annual basis, which increases the completeness and improves the quality of the information, as well as allowing the elimination of duplicate entries [18].

**Figure 1:**
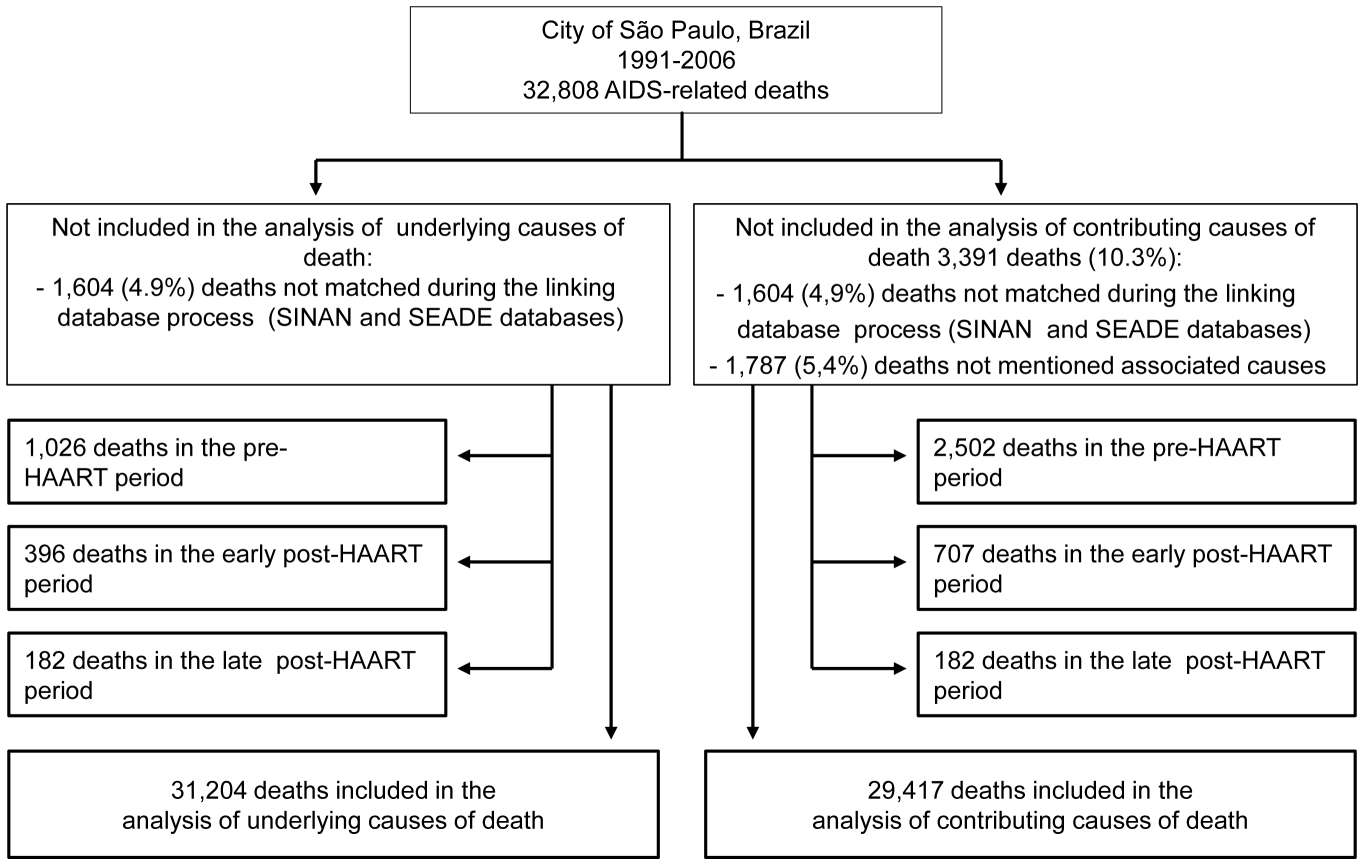
Flowchart of the grouping of deaths. Flowchart of the grouping of deaths included in the analysis of underlying and associated causes of death. Notes: BIPAIDS: *Base Integrada Paulista de AIDS* (São Paulo State Integrated AIDS Database); HAART: highly active antiretroviral therapy.

During the study period as a whole, 32,808 deaths were included in the BIPAIDS. As can be seen in Fig. 1, we selected 31,204 (95.1%) deaths for which the BIPAIDS contained information on the underlying and among them 29,417 (94.3%) for which were available data related to the associated cause(s) of death.

The study variables were sociodemographic characteristics (gender, age, and area of residence), underlying causes of death, associated causes of death, and socioeconomic characteristics of the districts of residence. For the processing of the underlying and associated causes of death, we used the Multiple Causes of Death Tabulator developed by Santo & Pinheiro [13].

We analyzed data for three periods: pre-HAART (1991–1996); early post-HAART (1997–1999); and late post-HAART (2000–2006). In order to characterize the study population and provide evidence of changes in the patterns of mortality in the three periods evaluated, we present, for each period, the proportional distribution of the underlying causes of death, and the associated causes of death. Comparative analysis of categorical variables was performed with the Pearson's chi-square test or Fisher's exact test, as appropriate, or with the chi-square test for trend, when the data suggested a linear trend.

Mortality rates were based on aggregate data. We calculated those rates using the number of deaths as the numerator and the population (estimated at mid-year) as the denominator. The mortality rates for the main underlying causes of death were estimated for the 1996–2006 period and adjusted for age by the direct method, the population of the city of São Paulo in 2000 (as estimated by the 2000 Brazilian Demographic Census) being used as a reference. In order to avoid the use of the ICD-9 and ICD-10 in the same historical series, we did not estimate the mortality rates for the main underlying causes of death for the 1991–1995 period.

To analyze trends in groups of selected causes of death, we used a generalized linear model with Poisson regression. In evaluating the goodness-of-fit of the model, we considered the parameters coefficient of determination (r^2^) and statistical significance (p <0.05).

To describe the changes in the underlying and associated causes of death, we compare the proportional distribution of each underlying and associated cause of death in relation to all AIDS-related deaths recorded in each period.

In order to analyze the districts of residence, we used a classification whereby the districts of the city of São Paulo are grouped into four homogeneous areas by means of the São Paulo State Social Vulnerability Index. The areas are as follows: predominantly high-income areas (17 districts); predominantly middle-income areas (35 districts); areas in transition to becoming middle-income (25 districts); and predominantly low-income areas (19 districts). The São Paulo State Social Vulnerability Index was developed by the São Paulo SEADE Foundation and is a composite index that takes into consideration the income, level of education, and age of the head of household, as well as whether there are children ≤ 4 years of age living in the household [19]. The analysis of the districts of residence was conducted for the 2000–2006 period, because the goal was to identify potential disparities in the late post-HAART period, as well as because of the operational difficulties related to changes in the delineation of the districts in the 1990s, which would have precluded the use of the social vulnerability index.

### Ethics Statement

The present study used a secondary, anonymized data set obtained from a public repository–the STD and AIDS Referral and Training Center–as well as from the São Paulo State STD/AIDS Program and the São Paulo State STD/AIDS Surveillance System (www.crt.saude.sp.gov.br). The study was approved by the Research Ethics Committees of the University of São Paulo School of Public Health (Protocol no. 2,163/2010) and the STD and AIDS Referral and Training Center (Protocol no. 008/2010), both of which are located in the city of São Paulo, and had ethical oversight from the institutional review board of the University of São Paulo School of Public Health and the STD and AIDS Referral and Training Center.

## Results

As of December 31, 2006, the number of AIDS cases among residents of the city of São Paulo reported to the São Paulo State AIDS Surveillance System had reached 64,964. Of the 64,964 affected individuals, 32,808 (50.5%) had died in the 1991–2006 period. Of those 32,808 individuals, 24,954 (76%) were male. In that period, there was an increase in the number of AIDS cases among females residing in the city of São Paulo. Between 1991 and 2006, the proportion of deaths among females increased from 14.3% (341 of the 2,383 deaths reported) to 31.0% (416 of the 1,341 deaths reported). The mortality rate increased continuously until 1994, stabilized in 1995 and began to decline, coincident with the introduction of HAART, in 1996. There was a 63.2% decrease in mortality (from 42.7/100,000 person-years in 1995 to 15.7/100,000 person-years in 2006).

Table 1 shows the descriptive data for the proportional distribution of the underlying and associated causes of death in the three periods evaluated. When we compared the first period (1991–1996) with the third period (2000–2006), we found that the proportion of deaths whose underlying causes were non-AIDS-related diseases increased from 0.2% (41 of 16,421 deaths reported) to 9.6% (918 of the 9,579 deaths reported), the difference between the two periods being significant (p <0.001). The annual mean mortality rates of non-AIDS-related causes adjusted for age also increased, from 0.1/100,000 person-years in the 1991–1996 period to 1.5/100,000 person-years in the 2000–2006 period (RR  =  17.86; 95%CI 8.34–38.23). As can be seen in Table 1, the underlying causes of death for which the proportion of death increased the most between the pre-HAART period and the late post-HAART period were cardiovascular disease (from 0.01% to 1.67%; p <0.001), bacterial/unspecified pneumonia (from 0.01% to 1.62%; p <0.001), and NADCs (from 0.03% to 1.46%; p <0.001). In the late post-HAART period, deaths whose underlying causes were ischemic heart diseases accounted for 50% (80) of the 160 deaths whose underlying causes were cardiovascular diseases. In addition, external causes were the second leading underlying causes of non-AIDS-related deaths, accounting for 2.93% (281) of the 9,579 deaths occurring in the late post-HAART period.

**Table 1:** Deaths of people ≥ 13 years of age living with AIDS, by underlying cause of death, associated cause of death, and period of death. São Paulo, Brazil, 1991–2006.

Underlying causes of death^1^	Period				Associated causes of death^1^	Period			
	**Pre-HAART**	**Early post-HAART**	**Late post-HAART**	**p** *		**Pre-HAART**	**Early post-HAART**	**Late post-HAART**	
	**1991–1996**	**1997–1999**	**2000–2006**			**1991–1996**	**1997–1999**	**2000–2006**	
	**n = 16,421**	**n = 5,204**	**n = 9,579**			**n = 14,945**	**n = 4,893**	**n = 9,579**	
	**n (%)** **	**n (%)** **	**n (%)** **			**n (%)** **	**n (%)** **	**n (%)** **	**p** *
**AIDS** (codes 279.1 and B20–B24)	**16,278 (99.13)**	**4,997 (96.02)**	**8,330 (86.96)**	<0.001	**Infectious and parasitic diseases** 2	11,530 (77.15)	4,432 (90.58)	8,782 (91.68)	<0.001
**Infectious and parasitic diseases** 2	**95 (0.68)**	**40 (0.77)**	**154 (1.61)**	<0.001	Tuberculosis	2,774 (18.56)	1,258 (25.71)	1,866 (19.48)	<0.001
*Pneumocystis jirovecii* pneumonia	26 (0.16)	1 (0.02)	8 (0.08)	0.107	Septicemia	2,172 (14.53)	1,104 (22.56)	3,205 (33.46)	<0.001
Tuberculosis	33 (0.20)	24 (0.46)	82 (0.86)	<0.001	CNS toxoplasmosis	2,036 (13.62)	649 (13.26)	968 (10.11)	<0.001
CNS toxoplasmosis	14 (0.09)	2 (0.04)	5 (0.05)	0.341	*P*. *jirovecii* pneumonia	1,682 (11.25)	463 (9.46)	743 (7.76)	<0.001
Cryptococcosis	11 (0.07)	1 (0.02)	3 (0.03)	0.279	Cryptococcosis	827 (5.53)	189 (3.86)	377 (3.94)	<0.001
Septicemia	2 (0.01)	3 (0.06)	16 (0.17)	<0.001	Viral hepatitis	70 (0.47)	111 (2.27)	547 (5.71)	<0.001
Viral hepatitis	1 (0.01)	2 (0.04)	20 (0.21)	<0.001	**Neoplasms** 2	**921 (6.16)**	**309 (6.32)**	**979 (10.22)**	<0.001
**Neoplasms** 2	**7 (0.04)**	**37 (0.71)**	**170 (1.77)**	<0.001	ADCs	700 (4.68)	200 (4.09)	515 (5.38)	0,010
ADCs	2 (0.01)	10 (0.19)	30 (0.31)	<0.001	NADCs	221 (1.48)	109 (2.23)	464 (4.84)	<0.001
NADCs	5 (0.03)	27 (0.52)	140 (1.46)	<0.001	**Diseases of the blood and blood-forming organs, and certain disorders involving the immune mechanism** 2	**415 (2.78)**	**201 (4.11)**	**545 (5.69)**	<0.001
**Endocrine, nutritional, and metabolic diseases** 2	**4 (0.02)**	**3 (0.06)**	**38 (0.40)**	<0.001	**Endocrine, nutritional, and metabolic diseases** 2	**767 (5.13)**	**399 (8.15)**	**967 (10.09)**	<0.001
Diabetes mellitus	2 (0.01)	3 (0.06)	25 (0.26)	<0.001	Diabetes mellitus	59 (0.39)	48 (0.98)	178 (1.86)	<0.001
**Diseases of the circulatory system** 2	**1 (0.01)**	**18 (0.35)**	**191 (1.99)**	<0.001	**Mental disorders** 2	**108 (0.72)**	**84 (1.72)**	**245 (2.56)**	<0.001
Cardiovascular diseases	1 (0.01)	16 (0.31)	160 (1.67)	<0.001	Alcohol use disorders	50 (0.33)	63 (1.29)	174 (1.82)	<0.001
Cerebrovascular diseases	0(0.00)	2 (0.04)	26 (0.27)	<0.001	**Diseases of the nervous system** 2	**1,574 (10.53)**	**506 (10.34)**	**810 (8.46)**	<0.001
**Diseases of the respiratory system** 2	**9 (0.05)**	**29 (0.56)**	**198 (2.07)**	<0.001	**Diseases of the circulatory system** 2	**550 (3.68)**	**331 (6.76)**	**1,168 (12.19)**	<0.001
Bacterial/unspecified pneumonia	1 (0.01)	20 (0.38)	155 (1.62)	<0.001	Cardiovascular diseases	445 (2.98)	272 (5.56)	968 (10.11)	<0.001
**Diseases of the digestive system** 2	**10(0.6)**	**25 (0.48)**	**85 (0.89)**	<0.001	Cerebrovascular diseases	105 (0.70)	59 (1.21)	200 (2.09)	<0.001
Liver diseases	7 (0.04)	21 (0.40)	56 (0.58)	<0.001	**Diseases of the respiratory system** 2	**10,681 (71.47)**	**3,686 (75.33)**	**7,284 (76.04)**	<0.001
**External causes** 2	**12 (0.07)**	**34 (0.65)**	**281 (2.93)**	<0.001	Bacterial/unspecified pneumonia	3,857 (25.81)	1.472 (30.08)	3.443 (35.94)	<0.001
Assault	1 (0.01)	24 (0.46)	135 (1.41)	<0.001	ARDS and emphysema	2,129 (14.25)	107 (2.19)	391 (4.08)	<0.001
Suicide	2 (0.01)	0(0.00)	28 (0.29)	<0.001	**Diseases of the digestive system** 2	**1,061 (7.10)**	**559 (11.42)**	**1,440 (15.03)**	<0.001
Accidents	6 (0.04)	6 (0.12)	68 (0.71)	<0.001	Liver diseases	331 (2.21)	271 (5.54)	766 (8.00)	<0.001
**Other causes**	**5 (0.03)**	**21 (0.40)**	**132 (1.38)**		**Diseases of the genitourinary system** 2	**425 (2.84)**	**282 (5.76)**	**818 (8.54)**	<0.001
					Renal failure	301 (2.01)	197 (4.3)	592 (6.18)	<0.001
					**External causes** 2	**37 (0.25)**	**84 (1.72)**	**639 (6.67)**	<0.001

Notes: HAART: highly active antiretroviral therapy; CNS: central nervous system; ADCs: AIDS-defining cancers; NADCs: non-AIDS-defining cancers; ARDS: acute respiratory distress syndrome.

1 The table shows only major underlying and associated causes of death rather than showing all of the conditions described in each chapter of the International Classification of Diseases; includes 31,204 and 29,417 deaths for which data regarding the underlying and associated causes, respectively, were available.

2 Including all underlying and associated causes of death described in each chapter of the International Classification of Diseases.

*Pearson's chi-square test or Fisher's exact test applied in order to compare the pre-HAART and late post-HAART periods.

**Proportions calculated in relation to the number of deaths in each period.

**Figure 2:**
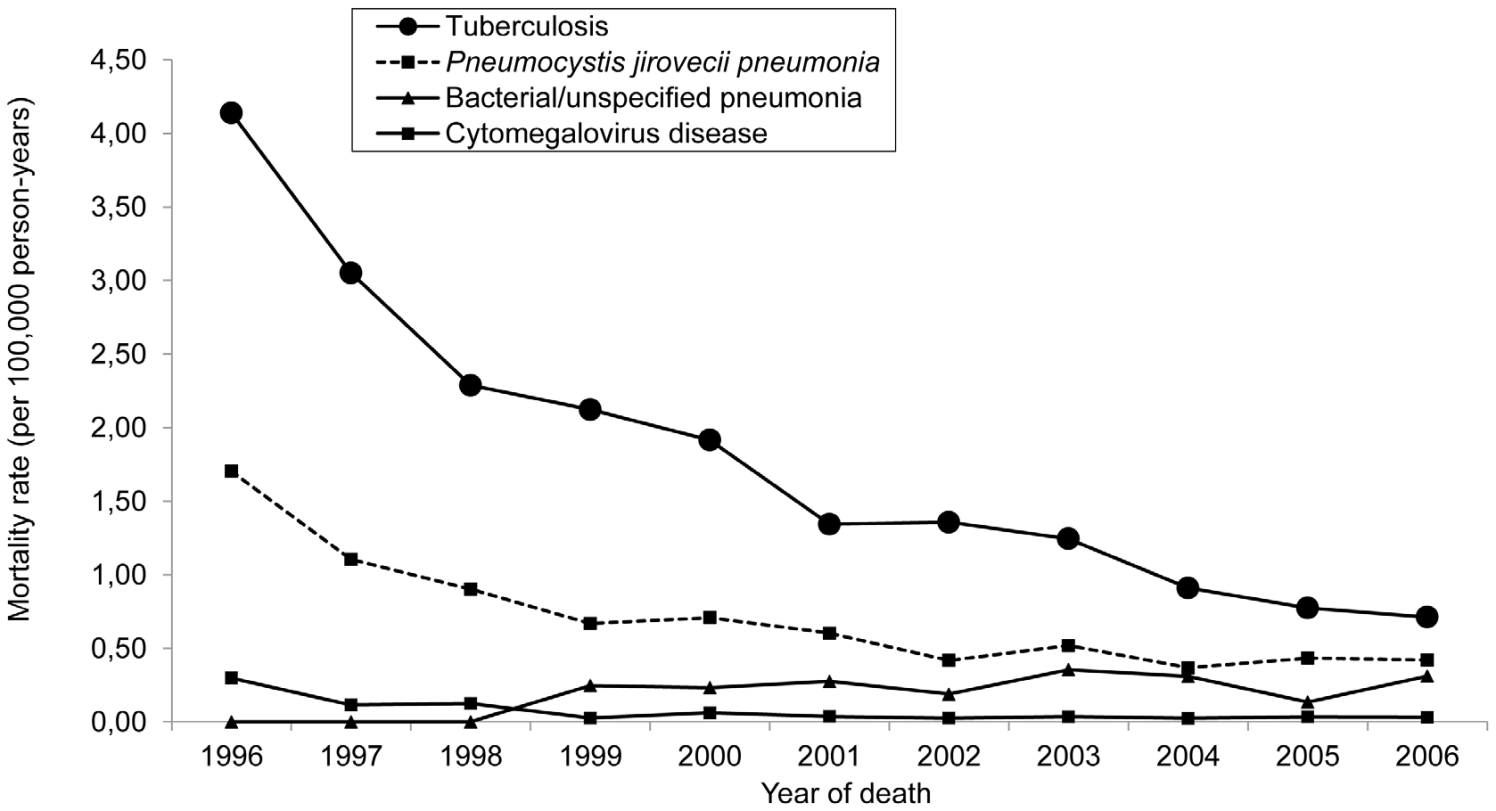
Mortality from opportunistic infections. Mortality rate (per 100,000 person-years and adjusted for age) for opportunistic infections as the underlying causes of death in people ≥ 13 years of age living with AIDS, by year of death. São Paulo, Brazil, 1996–2006.

**Figure 3:**
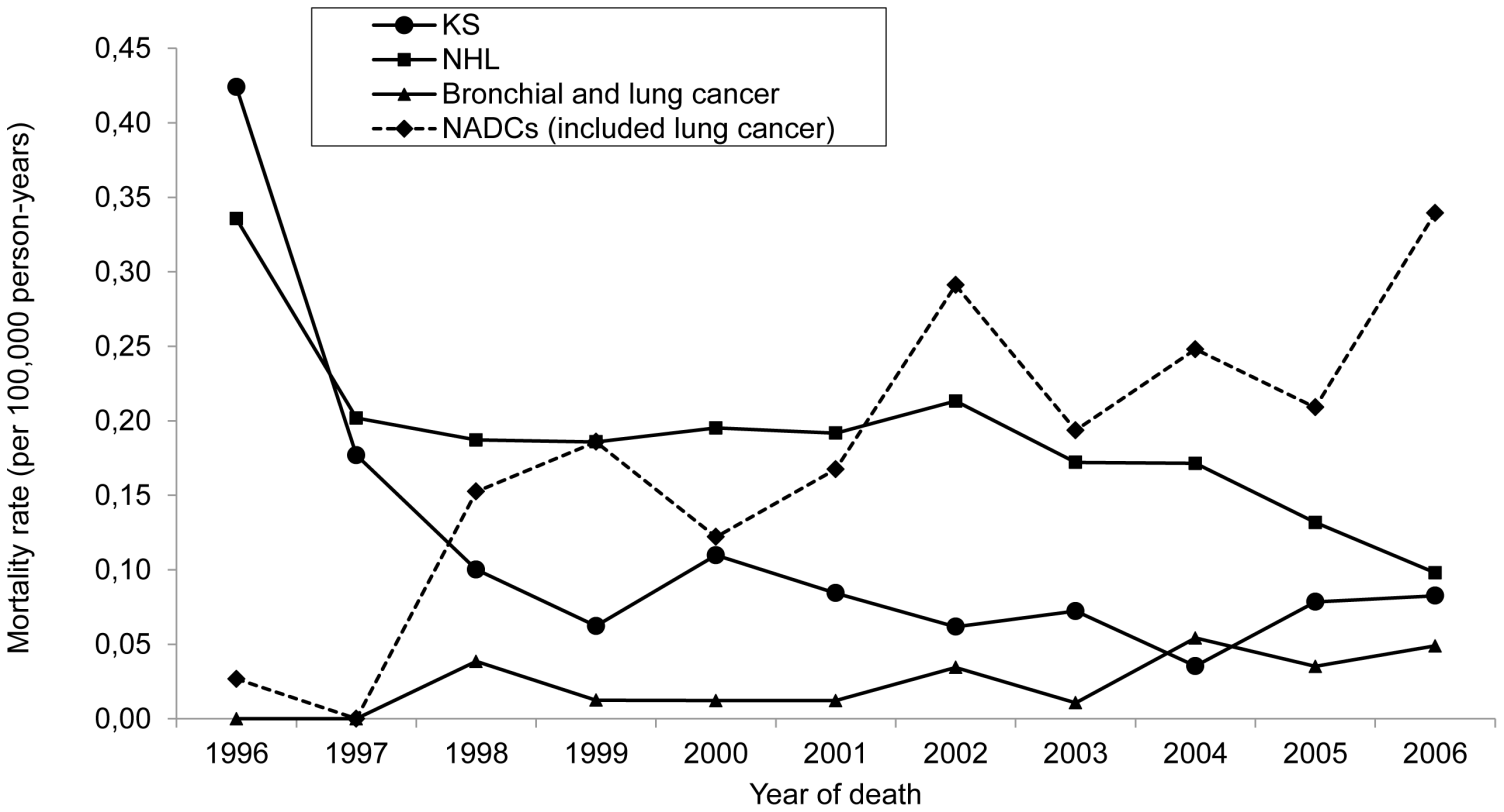
Mortality from cancers. Mortality rate (per 100,000 person-years and adjusted for age) for cancers as the underlying causes of death in people ≥ 13 years of age living with AIDS, by year of death. São Paulo, Brazil, 1996–2006. Notes: KS: Kaposi's sarcoma; NHL: non-Hodgkin's lymphoma; NADCs: non-AIDS-defining cancers.

Fig. 2 shows our analysis of trends of deaths from major opportunistic infections after the introduction of HAART (1996–2006). We found a decreasing trend in the rate of mortality from *Pneumocystis jirovecii* pneumonia (from 1.70/100,000 person-years in 1996 to 0.42/100,000 person-years in 2006, corresponding to a 75.3% decrease–RR  =  0.86; r^2^  =  0.61; p <0.0001) and in that of mortality from tuberculosis (from 4.14/100,000 person-years in 1996 to 0.71/100,000 person-years in 2006, an 82.8% decrease–RR  =  0.88; r^2^  =  0.86; p <0.0001). In addition, we found an 80.5% decrease in mortality from Kaposi's sarcoma (from 0.42/100,000 person-years in 1996 to 0.08/100,000 person-years in 2006–RR  =  0.83; r^2^  =  0.34; p <0.0001) and a 70.8% decrease in mortality from non-Hodgkin's lymphoma (from 0.34/100,000 person-years in 1996 to 0.10/100,000 person-years in 2006–RR  =  0.93; r^2^  =  0.12; p  =  0.006). In contrast, as shown in Fig. 3, mortality from NADCs increased by 12.7 times (from 0.03/100,000 person-years in 1996 to 0.34/100,000 person-years in 2006–RR  =  1.20; r^2^  =  0.37; p <0.0001). Among the deaths that occurred in the late post-HAART period and whose underlying causes were NADCs, the most common were those caused by bronchial or lung cancer (accounting for 13.6%); malignant neoplasms of the lip, oral cavity, or pharynx (accounting for 10%); and stomach cancer (accounting for 10%).

**Figure 4:**
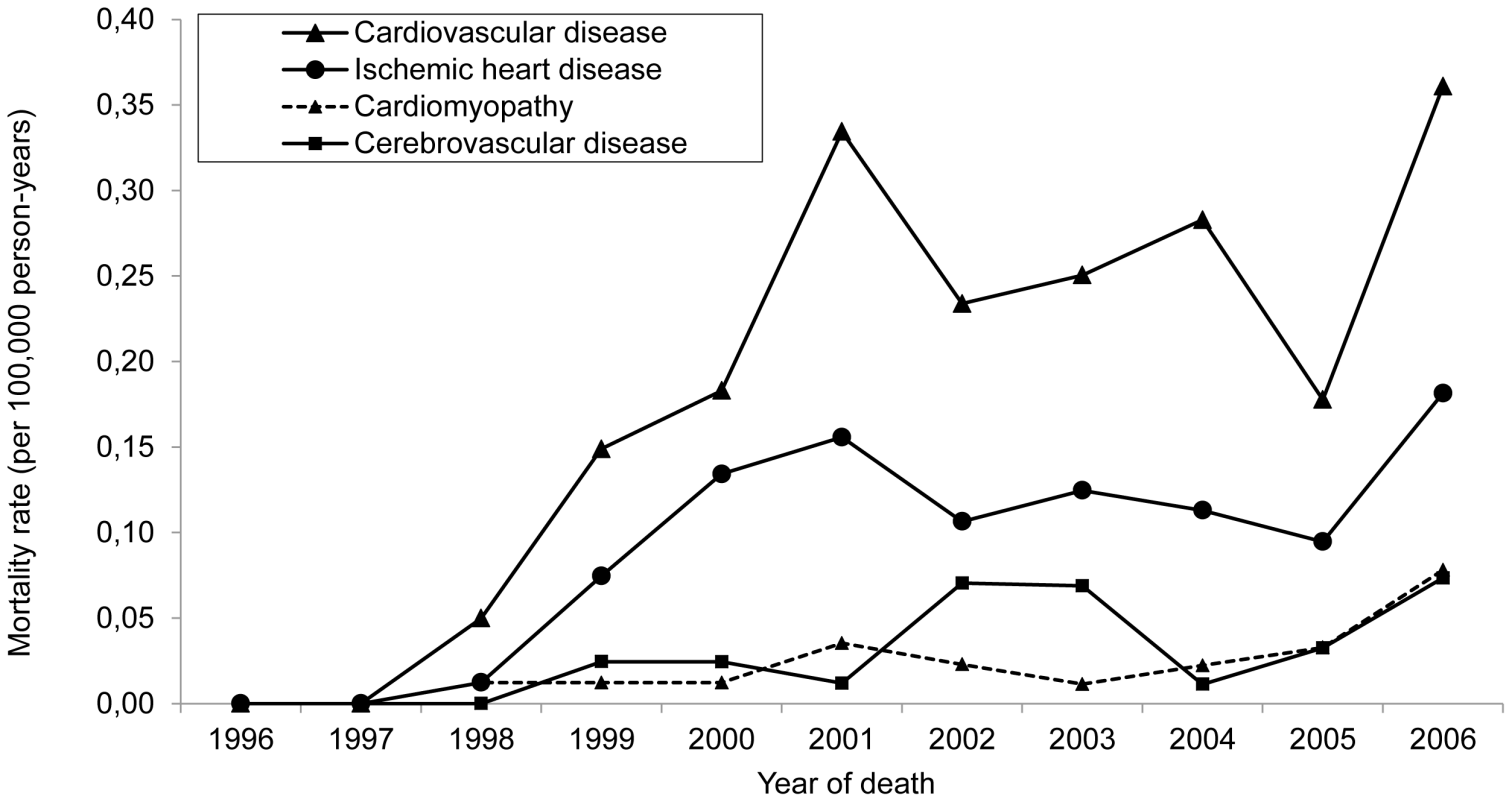
Mortality from diseases of the circulatory system. Mortality rate (per 100,000 person-years and adjusted for age) for diseases of the circulatory system as the underlying causes of death in people ≥ 13 years of age living with AIDS, by year of death. São Paulo, Brazil, 1996–2006.

The first records of deaths from cardiovascular and cerebrovascular diseases as the underlying causes of death occurred in 1998 and 1999, respectively. Mortality from cardiovascular diseases increased by 7.2 times (from 0.05/100,000 person-years in 1998 to 0.36/100,000 person-years in 2006–RR  =  1.31; r^2^  =  0.30; p  =  0.001).Chief among the cardiovascular diseases were ischemic heart disease and cardiomyopathy (Fig. 4). Between 1998 and 2006, mortality from ischemic heart disease increased by 14.4 times (from 0.01/100,000 person-years to 0.18/100,000 person-years–RR  =  1.2; r^2^  =  0.30; p <0.0001), whereas mortality from cardiomyopathy increased by 6.3 times (from 0.01/100,000 person-years to 0.08/100,000 person-years–RR  =  1.3; r^2^  =  0.30; p  =  0.001). Mortality from cerebrovascular diseases also increased by 3.0 times (from 0.02/100,000 person-years in 1999 to 0.07/100,000 person-years in 2006–RR  =  1.3; r^2^  =  0.25; p  =  0.001).

In the late post-HAART period, the leading underlying causes of death (other than AIDS), by gender, were cardiovascular disease and assault in males; and bacterial/unspecified pneumonia and NADCs in females. Among the 665 deaths reported in individuals 50 years of age or older, cardiovascular diseases and NADCs accounted for 59 (3.5%) and 57 (3.4%), respectively.

Table 1 shows the changes in the associated causes of death in all three of the periods analyzed. Comparing the pre-HAART and late post-HAART periods, we found that the proportion of deaths from septicemia increased from 14.53% to 33.46% (p <0.001); the proportion of deaths from bacterial/unspecified pneumonia increased from 25.81% to 35.94% (p <0.001); the proportion of deaths from cardiovascular disease increased from 2.98% to 10.11% (p <0.001); and the proportion of deaths from liver disease increased from 2.21% to 8.0% (p <0.001). Among all deaths analyzed in the present study, approximately 96.2% of the death certificates (30,030/31,204) included the term AIDS (or its equivalent in Brazilian Portuguese), or related terms. In the pre-HAART period, that proportion was nearly 100.0% (16,414 of the 16,421 deaths reported). However, in the late post-HAART period, there was a reduction of 10.7% in the number of death certificates mentioning AIDS; that is, of the 9,579 death certificates issued during that period, there were 1,021 that contained no mention of AIDS. We were able to identify deaths from non-AIDS-related causes and deaths in which AIDS was (intentionally or unintentionally) not mentioned on the death certificate because the linkage from the death database of the state of São Paulo (SEADE, State System of Data Analysis) and the SINAN Reportable Diseases Database (São Paulo State Program for STDs and AIDS).

Underreporting of AIDS on the death certificate occurred in a total of 1,021 cases and was more common among death certificates for deceased males than among those for deceased females, occurring in 792 (11.8%) of 6,731 cases and 229 (8.0%) of 2,848 cases, respectively (p <0.001). Among those 1,021 deaths, the main underlying causes of death listed were bacterial/unspecified pneumonia, in 155 (15.2%); assault, in 135 (13.2%); cardiovascular disease, in 118 (11.6%); tuberculosis, in 82 (8.0%); NADCs, in 46 (4.5%); suicide, in 27 (2.6%); and cerebrovascular disease, in 19 (1.9%).

In the late post-HAART period, the proportion of deaths whose underlying causes were non-AIDS-related diseases was higher in predominantly high-income areas than in predominantly low-income areas (11.9% vs. 8.6%; p  =  0.004). The leading underlying causes of death (other than AIDS), unevenly distributed by place of residence, were as follows: NADCs, in predominantly high-income areas; cardiovascular diseases, in predominantly middle-income areas; and assault, in predominantly low-income areas. In addition, among the associated causes of death in the late post-HAART period, tuberculosis and AIDS-defining cancers varied significantly across the areas of residence. Tuberculosis was an associated cause of death in 14.7%, 18.6%, and 20.6% of the deaths occurring in predominantly high-income areas, predominantly middle-income areas, and predominantly low-income areas, respectively (p <0.001 for linear trend). In addition, AIDS-defining cancers were associated causes of death in 9.3%, 5.5%, and 4.3% of the deaths occurring in predominantly high-income areas, predominantly middle-income areas, and predominantly low-income areas, respectively (p <0.001 for linear trend).

## Discussion

The results of the present study show that there have been changes in the mortality profile of people living with AIDS in the city of São Paulo, such changes having occurred after the introduction of HAART and possibly reflecting changes in the comorbidity profiles of those patients [5], [7]. This trend is similar to that observed in industrialized countries [20], [21] and is perfectly explained by the success of the Brazilian policy for universal access to diagnosis and treatment [22], [23] and by the highly favorable results of the AIDS control program in the city of São Paulo, where there was a decrease of approximately 65% in AIDS mortality in the post-HAART period.

Among the most relevant findings of the present study was the nearly 15-fold increase in the rate of mortality from non-AIDS-related diseases between the pre-HAART period and the late post-HAART period, principally from cardiovascular disease, bacterial/unspecified pneumonia, and NADCs. Chief among the associated causes of death in the late post-HAART period were bacterial/unspecified pneumonia, septicemia, cardiovascular disease, and liver disease. Finally, it is of note that such changes in the mortality profile were not homogeneous across the various districts of the city of São Paulo, NADCs being more common in predominantly high-income areas, cardiovascular diseases being more common in predominantly middle-income areas, and assault being more common in predominantly low-income areas. The differences among the places of residence reflects the social disparities in the city of São Paulo, where individuals living in districts with better socioeconomic conditions have a mortality profile that is similar to that of individuals living in developed countries, whereas those living in poorer areas have a profile comparable that of other developing countries; this is indicative of the social inequalities that exist in the city, which are not remedied by the policy of distributing antiretroviral drugs at no cost to the patients [4], [24].

Various studies, including studies conducted in Brazil, have shown an increase in cardiovascular disease as a cause of death in people living with HIV/AIDS [5], [25]–[27]. The impact of cardiovascular disease can be greater in patients with HIV/AIDS than in the general population [27], [28], principally because of the use of antiretroviral therapy (particularly protease inhibitors), which can contribute to the development of metabolic dysfunction and lipodystrophy, or because of the effects of HIV infection itself [29]–[32]. In the present study, ischemic heart disease accounted for half of the deaths from cardiovascular disease.

Our finding of a reduction in opportunistic infections, particularly *Pneumocystis jirovecii* pneumonia, is consistent with data in the literature [20], [26]. In addition, our finding of an increase in the proportions of bacterial/unspecified pneumonia and septicemia as underlying and associated causes of death in the late post-HAART period is similar to those of studies conducted in developed countries [26], [33]. Some authors have reported that bacterial infections are more common among patients in developing countries [4]. This difference is attributed to situations that cause severe impairment of the immune system, including late diagnosis of HIV infection, lack of use of or poor adherence to antiretroviral therapy, and initiation of HAART in the presence of opportunistic infections [34].

Increased survival, contributing to an increase in the mean age of patients on HAART, has led to the emergence of cancers, especially NADCs [35], . Among malignancies, lung cancer was found to be the most common underlying cause of death in the late post-HAART period, a finding that is consistent with those of other studies [9], [24], [37]. The most widely accepted hypothesis is that HIV-infected individuals are at a high risk for lung cancer because of excessive smoking and other factors that can act synergistically with exposure to tobacco smoke, such as frequent pulmonary infection or inflammation [24]. The presence of cancers of the lip, oral cavity, pharynx, rectum, anus, or anal canal suggests that oncogenic viruses, such as human papillomavirus, are involved in such events [36]; like observed in other Brazilian study for anal intraepithelial lesions [38].

Among non-AIDS-related causes of death in the late post-HAART period, the proportion of deaths from external causes in the city of São Paulo was found to be quite high, about one-third. During a similar period (2000–2007), that proportion was only approximately 7% nationwide [25], however, these causes have been growing in HIV-infected population in the country [27]. This might be due to the characteristics of the study population, because we found that, during that same period (2000–2007), the proportionate mortality from such causes dropped from 15% to 10% in the city of São Paulo (unpublished data: São Paulo *Sistema Estadual de Análise de Dados* (SEADE) Available: http://www.seade.gov.br/produtos/imp/index.php. Accessed 12 May 2014), which is consistent with the findings of other recent studies [39]. However, it is possible that it is more closely related to factors present in large urban areas, including violence, heavy traffic, a high number of inhabitants, and poor living conditions.

The increase in the number of deaths from non-AIDS-related causes was at least partially responsible for AIDS having been omitted from 10.7% of the death certificates issued during the late post-HAART period; similar situation was also identified in the city of Rio de Janeiro, Brazil, after the introduction of HAART [40]. However, we cannot rule out the possibility that other factors, such as confidentiality and the social stigma of AIDS, played a role [41].

The results of the present study should be interpreted in light of several limitations, including, first and foremost, the use of secondary data, as well as the quality of the information obtained from death certificates and the coding of the causes of death, which can lead to classification errors. The use of two revisions of the International Classification of Diseases, i.e., ICD-9 and ICD-10, could lead to errors because of the changes that were made in the latter revision, limiting the comparability of the results [42]. However, such errors were probably minimized by the use of tables of equivalence between ICD-9 and ICD-10 in the present study [14]. Another limitation of our study is that our data were subject to changes over time in the definition of an AIDS case, especially because the use of the earliest definitions might have resulted in underdiagnosis and a consequent underreporting of cases.

Despite its limitations, the present study produced results that are consistent with the literature and provides data to support public policies and clinical practices aimed at preventing and treating diseases resulting from HIV infection in this new context of altered morbidity and mortality. In addition, our findings indicate the need for studies aimed at identifying factors that explain the differences in causes of death among the areas of the city of São Paulo and provide data to support interventions focusing on promoting health equity. Although measures to reduce AIDS mortality entail increased expenditures, they are needed for an overall improvement in the health care services provided to people living with HIV/AIDS; for early diagnosis of HIV infection; for timely initiation and proper use of HAART; and for improving the efficacy and tolerability of currently available antiretroviral therapies, as well as for surveillance of any adverse effects of those therapies.
